# High-performance classification of contour percepts from EEG recordings

**DOI:** 10.1186/1471-2202-12-S1-P94

**Published:** 2011-07-18

**Authors:** David Rotermund, Marc Schipper, Manfred Fahle, Udo A Ernst

**Affiliations:** 1 Institute for Theoretical Physics, Cognium, Hochschulring 18, University of Bremen, Bremen, D-28359, Germany; 2 Dept. for Human Neurobiology, Cognium, Hochschulring 18, University of Bremen, Bremen, D-28359, Germany

## 

**Figure 1:**
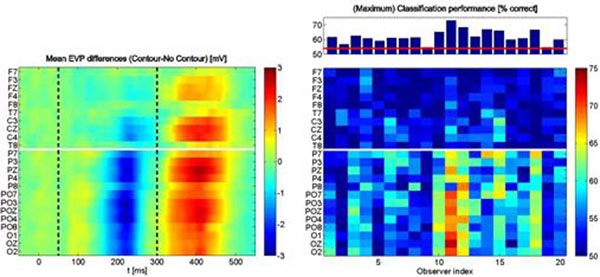
The left panel shows the difference of the evoked visual potentials (EVPs) for contour stimuli versus non-contour stimuli, averaged over the observers. First signatures of contour integration processes appear at around 180 ms after stimulus onset in the occipital-parietal electrodes (below the white line), followed by the frontal electrodes at about 220 ms (P200), lasting about 70 ms. EVP differences with the opposite sign appear from about 320 ms and extend for about 140 ms. Mean reaction time was around 550 ms, with large variations across observers. Differences in EVPs occurring after 300 ms (i.e., ~200 ms before the observer's responses) are likely to reflect mixtures of late contour integration and early motor preparatory processes. These periods were thus excluded from classification, using only wavelet coefficients from 50 to 300 ms after stimulus onset (black dashed line). The right panel shows classification performances. With a chance level at 50% and an error probability of 2%, performances >53.8% are considered significantly different from chance level. Performance varies considerably over observers, and is highest in the parietal-occipital electrodes (right panel), for wavelet frequencies between 3-4 Hz (not shown).

Contour integration is a fundamental process for visual scene segmentation and object recognition. Consequently, human observers are very efficient in detecting configurations of aligned edge elements in a background of randomly oriented distracters. Neural signatures of contour integration processes have been found in electrophysiological recordings in the early visual areas of primates, and in EEG signals from the occipital areas in human subjects. However, the corresponding differences in the signals between stimuli containing contours or no contours are normally small, and only show up after extensive averaging over trials. In this contribution, we investigate neural signatures of contour integration processes in EEG recordings by classifying the presence or absence of contours on a trial-by-trial basis from the recorded data. Stimuli consisted of fields of oriented Gabor elements, which were positioned randomly on the screen. Half of the stimuli contained an elliptic contour, which was formed by 13 colinearily aligned edges. In a two-alternative-forced-choice task, 20 observers had to indicate the presence or absence of a contour by pressing a corresponding response button. To our surprise, classification performance on the EEG data can be as high as 78% in single observers, averaging at about 64% over our 20 observers. Given that all stimuli have the same number of ~350 Gabor elements and only differ in the alignment of a small subset of 13 edges, differences in the EEG and in its classification reflect differences between perceptual states, rather than differences between physical stimuli. In the context of constructing EEG-based brain-computer interfaces, these perceptual differences may serve as an additional channel of information for paradigms like SSVEPs which otherwise use different visual stimuli to evoke maximally distinct brain activity patterns. Figure 1.

